# The Effect of High-Intensity Accelerations and Decelerations on Match Outcome of an Elite English League Two Football Team

**DOI:** 10.3390/ijerph18189913

**Published:** 2021-09-21

**Authors:** David Rhodes, Stephen Valassakis, Lukasz Bortnik, Richard Eaves, Damian Harper, Jill Alexander

**Affiliations:** 1 Football Performance Hub, Institute of Coaching and Performance (ICaP), School of Sport and Health Sciences, University of Central Lancashire, Preston PR1 2HE, UK; lukbor@hotmail.com (L.B.); DHarper5@uclan.ac.uk (D.H.); jalexander3@uclan.ac.uk (J.A.); 2 Sport, Nutrition and Clinical Sciences, School of Sport and Health Sciences, University of Central Lancashire, Preston PR1 2HE, UK; StephenV@PhysFitRehab.com (S.V.); rseaves@uclan.ac.uk (R.E.)

**Keywords:** soccer, conditioning, high velocity actions, performance, injury risk reduction

## Abstract

Objectives: Previous research has highlighted the frequency of high-intensity accelerations and decelerations in elite football. The influence of these actions on match performance outcomes has not been established. The aim of the present study was to identify the influence of high-intensity accelerations and decelerations on match performance outcomes (i.e., win, draw, lost). Comparisons were also made between team and positional high-intensity accelerations and decelerations recorded within the games. Methods: 26 elite outfield footballers from an elite English Football League (EFL) Two team completed the present study. Global Positioning System (GPS) technology was utilised to quantify high-intensity accelerations and decelerations during 45 games in a competitive season. Magnitude analysis and the effects of results, positions and fixture periods were observed. Results: Significant effects of results, periods and positions were observed (*p ≤* 0.05), with the highest outputs observed in games won. Positionally, fullbacks and centre forwards in a 4–3–3 formation exhibited the greatest frequency of high-intensity accelerations and decelerations. Very large differences were observed between the frequency of high-intensity decelerations compared to accelerations in games won (g = 2.37), drawn (g = 2.99) and lost (g = 3.59). The highest team frequencies of high-intensity accelerations (*n* = 3330) and decelerations (*n* = 6482) were completed in games won. Conclusions: The frequency of high-intensity accelerations and decelerations has a significant impact on match performance outcomes in an elite English League Two football team. Consideration needs to be given to specific conditioning and recovery strategies to optimise high-intensity acceleration and deceleration performance in games. Caution should be taken as these findings are representative of one team within the EFL.

## 1. Introduction

The high-performance sport of football and its demand for optimum levels of physical, technical, tactical and mental ability from players is well established [1,2]. Football performance involves prolonged aerobic activity with frequent intermittent bouts of unpredictable, high-intensity actions such as sprints, accelerations, decelerations, changes of direction, jumps and tackles [1,3]. Contemporary motion-tracking technologies are now used to quantify the mechanical impacts and corresponding physiological responses of high-intensity actions and thus aid to quantify and monitor player performance and fatigue [4]. Available evidence also supports the design and implementation of position-specific training programs, recovery and periodisation strategies to maximise performance, minimise fatigue and reduce injury risk [5,6]. Such factors are considered critical in the management of player match and training load, especially between periods of competitive fixture congestion that could increase injury risk and cause disruption to technical and tactical performance, both of which could ultimately impact team match performance and final league standing [7,8,9,10].

Of recent interest is the effect of high-intensity accelerations and decelerations on the physiological and mechanical demands and performance outcomes of players [11,12]. This contrasts with the previous focus on metrics of total distance and high-intensity running that when used in isolation fail to accurately quantify player exposure and could potentially lead to underestimation of player external and internal load demands [3,13,14]. This is particularly important when considering high-intensity accelerations and decelerations, where players may be exposed to significant levels of physiological and mechanical stress which could subsequently impact match performance and the post-match recovery timeline [6,12,14,15]. It is important to note that match performance is not restricted to match outcome. Contextual factors, like playing position, can influence other performance metrics such as physical performance outputs [16]. Accelerations and decelerations have been reported to contribute to between 5 and 10% of the total player load during competitive matches in elite Norwegian football [15]. The contribution of those metrics to understanding performance in elite English football, however, is not well described in the current literature. Such high-intensity accelerations and decelerations require significantly high rates of force development (particularly eccentric muscle actions) accompanied by rapid and highly coordinated neural activations [17]. Consequently, these actions may contribute to induced muscle damage and reduced neural drive and mechanical fatigue with potential detrimental effects on performance outcomes [6,13,15,18].

The influence of high-intensity decelerations on player load demands during match play is of particular note as high eccentric braking actions seem to account for the highest magnitude of mechanical load per metre than any other match play locomotor activity [14]. Accordingly, decelerations may induce high-volume eccentric muscle actions and have a cumulative detrimental effect on various performance markers following match play [17,19,20,21]. These include increased muscle soreness, reduced concentric/eccentric phase performance during countermovement jumps and isometric hamstring strength asymmetries. That said, current literature fails to analyse how the occurrence of both high-intensity decelerations and accelerations may influence match performance outcomes. Furthermore, the ‘cascade-effect’ on both athlete and team performance cannot be understated due to the potential of temporal and cumulative fatigue negatively impacting player performance and increasing susceptibility to injury [22]. Indeed, literature supports the utility of these metrics as sensitive markers of fatigue in football. For example, declines in the frequency and intensity of accelerations and decelerations have been noted during the second half of match plays [5,12,14,23]. Despite this, there is currently no research investigating the utility of high-intensity accelerations and decelerations as sensitive indicators of team performance.

Despite advancements in periodisation and injury prevention practices in elite sports, incidence rates of non-contact injuries in elite football have not improved [24]. To our knowledge, there has been little investigation on the effect of high-intensity accelerations and decelerations on match outcome, especially when comparing mid-week fixtures, Saturday games following a mid-week fixture and Saturday games in isolation which are not preceded by mid-week games. This is particularly relevant in contemporary elite football when teams are subjected to congested fixture periods and are high performance, which, coupled with fatigue management, are considered critical requirements for successful match performance outcomes [8,25]. This necessitates a wider investigation into how differences in the frequency of high-intensity accelerations and decelerations could potentially impact match outcome and how this may change in micro-cycles with one versus twice-weekly matches. Such findings could enhance current load periodisation practices and optimise recovery strategies that facilitate maximal team performance and success across a season. Thus, the aim of the present study was to examine the effect of high-intensity accelerations and decelerations on match outcome (i.e., win, lose or draw) in an elite English Football League Two team. Comparisons were also made between the frequency of both team and positional high-intensity accelerations and decelerations recorded within the games. It was hypothesized that increased acceleration and deceleration team outputs would have a positive effect on match outcome.

## 2. Materials and Methods

### 2.1. Participants

Twenty-six elite outfield football players from an English Football League team (mean ± SD: 23.68 ± 7.12 years, 183.4 ± 6.82 cm, 80.70 kg ± 6.42) participated in the study during the 2019/2020 season. The sample included five centre forwards, four centre halves, seven central midfielders, five fullbacks and five wingers. The formation of the team was consistent throughout all games analysed and represented a 4–3–3. In total, 57 competitive games were completed in this season. Games where formation was changed from the 4–3–3 were excluded (*n* = 12) to minimise the effect of formation on physical demands. Players were presented with information of the project protocol and provided informed consent for the use of match data in accordance with the Helsinki Declaration. All data was anonymised prior to data analysis to ensure player confidentiality. Ethical approval was provided by the host university (STEMH 0094).

### 2.2. Protocol

External workload data was collected from competitive matches (*n* = 45) using global positioning analysis (GPS) units (Catapult Optimeye S5, Catapult Innovations, Melbourne, Australia) to provide data at 10 Hz. Mean high-intensity accelerations and decelerations were derived from PlayerLoad™ instantaneously. Reliability of this system was previously reported, indicating excellent intra-device reliability. ICC values ranged from 0.77 (95% CI: 0.62–0.89) (very large) to 1.0 (95% CI: 0.99–1.0) (nearly perfect) [26]. The units were fitted securely into the back of the custom-made vests, positioned centrally on the upper back, with no restriction to players’ upper torso and limb range of motion. Specific units were assigned to players for the duration of the season as recommended to maximise the validity of obtained data. All matches were played on outdoor grass pitches in accordance with the English Football Association. Corresponding scores for each match were collected and categorised as either a win, loss or draw. Data were obtained from matches and categorised according to the period in which games occurred (i.e., Saturday game in isolation, mid-week game, typically a Tuesday evening, and Saturday game that followed a mid-week game) [9]).

Physical parameters of interest which were analysed in this study included frequency of accelerations and decelerations for each player during matches. Pre-determined thresholds criteria of >3 m/s^−2^ for accelerations and <−3 m/s^−2^ for decelerations were respectively utilised [14].

### 2.3. Data Analysis

Data were collected for analysis from each GPS unit, including accelerations >3 m/s^2^ and decelerations <3 m/s^2^. Following each game, data were downloaded into Catapult, Australian specialised analysis software (Openfield, 1.21.1). Occurrence of accelerations and decelerations above or below the relative thresholds set were extracted for mid-week games, Saturday games following a mid-week game and Saturday games in isolation that were not preceded with a mid-week game across the season and utilised for analysis.

### 2.4. Statistical Analysis

Differences in total team frequency of high-intensity accelerations and decelerations across matches with win, draw and lost performance outcomes were examined using Hedges’ *g* corrected effect size with 95% confidence intervals (95% CI), calculated using an online statistical spreadsheet [27] and interpreted using the scale from Hopkins [28] as: trivial (0.00 to 0.19), small (0.20 to 0.59), moderate (0.60 to 1.19), large (1.20 to 1.99), very large (2.0 to 4.0) and extremely large (>4.0).

Further statistical analyses were conducted using IBM Statistical Package for the Social Sciences (SPSS, Version 27.0, IBM Corporations, New York, NY, USA) with the statistical significance accepted at the 0.05 level. A univariate analysis of variance (ANOVA) was conducted to quantify main effects for result, period and position. Interaction effects were also quantified and any significant main effects associated with result, period and position were explored using post hoc pairwise comparisons with a Bonferroni correction factor. The assumptions associated with the statistical model were assessed to ensure model adequacy. To assess residual normality for each dependant variable, Q–Q plots were generated using stacked standardised residuals. Scatterplots of the stacked unstandardised and standardised residuals were also utilised to assess the error of variance associated with the residuals. Mauchly’s test of sphericity was also completed for all dependent variables, with a Greenhouse–Geisser correction applied if the test was significant. Partial eta squared (*η*^2^) were calculated to estimate effect sizes for all significant main effects and interactions. As recommended by Cohen [29], partial eta squared was classified as small (0.01–0.059), moderate (0.06–0.137) and large (>0.138).

## 3. Results

Table 1 below details the total number of accelerations and decelerations performed >3 m/s^2^ and <−3 m/s^2^ for each game type (midweek game (*n* = 15), Saturday following a midweek game (*n* = 15) and a Saturday game where no midweek game preceded it (*n* = 15)). The mean and standard deviation (SD) are reported for each game type. The total number of accelerations and decelerations performed (>3 m/s^2^ and <−3 m/s^2^) are reported for all games along with mean and SD for all games (*n* = 45). Further to this, Table 2 details the total, mean and SD values for accelerations and decelerations >3 m/s^2^ and <−3 m/s^2^, respectively, in relation to game type-highlighting outputs for wins, draws or losses. Finally, Table 3 details positional mean and SD within a 4–3–3 formation for accelerations and decelerations (>3 m/s^2^ and <−3 m/s^2^) over 45 competitive League Two fixtures.

**Figure 1:**
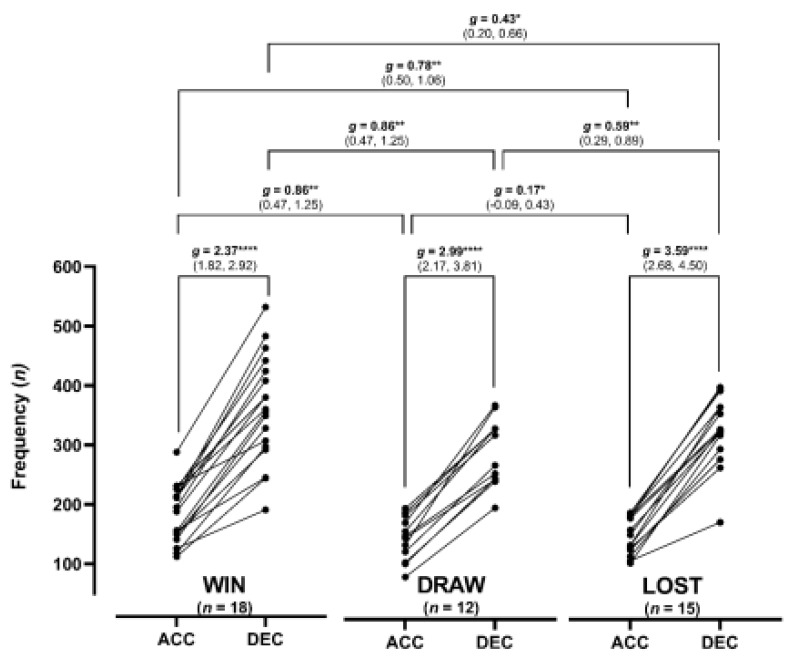
Total team frequency and effect size differences in high-intensity accelerations and decelerations completed during matches with win, draw and lost performance outcomes. Effect size differences displayed as: * = small, ** = moderate, **** = very large.

Total team frequency and effect size differences between high-intensity accelerations and decelerations completed during matches with win, draw and lost performance outcomes are presented in Figure 1. Regardless of match performance outcome, there was a very large difference (*g* = 2.37–3.59) between the frequencies of high-intensity decelerations compared to accelerations, with the greatest difference observed in matches lost (*g* = 3.59). The highest total team frequency of both high-intensity accelerations and decelerations were completed during matches won (185 ± 48 and 360 ± 90) in comparison to games drawn (146 ± 37 and 291 ± 55) and lost (152 ± 31 and 326 ± 59), respectively. Trivial-to-moderate differences (*g* = 0.17–0.86) were observed between high-intensity accelerations during matches with win, draw and lost performance outcomes. Similar effect size differences were also observed when comparing the frequency of high-intensity decelerations between matches won, drawn and lost (*g* = 0.43–0.86).

Analysis of accelerations and decelerations over 45 games in the season identified significant effects for result (Accelerations: *F* = 2.286, *p* = 0.01, *η*^2^ = 0.14; Decelerations: *F* = 1.474, *p* = 0.04, *η*^2^ = 0.08) and game type (Accelerations: *F* = 0.02, *p* = 0.03, *η*^2^ = 0.01; Decelerations: *F* = 0.125, *p* = 0.05, *η*^2^ = 0.01). There was a result *x* game type interaction for accelerations (*F* = 3.705, *p* = 0.01, *η*^2^ = 0.292), and no interaction was identified for decelerations (*p* > 0.05). The data set was collapsed to consider the result and game type in isolation. Significant differences were identified between wins and draws (Accelerations: *p* = 0.003; Decelerations: *p* = 0.01) and wins and losses (Accelerations: *p* = 0.01; Decelerations: *p* = 0.04) for both accelerations and decelerations. No significant differences were identified between draws and losses (*p* > 0.05). Significant differences were identified for accelerations from mid-week games to the Saturday following a mid-week game (*p* = 0.01) and mid-week games compared to Saturday games which were not preceded by a mid-week game (*p* = 0.01). Decelerations only displayed significant differences between mid-week games and the subsequent Saturday games (*p* = 0.002).

Positional analysis of accelerations and decelerations identified a significant effect of position (Accelerations: *F* = 4.490, *p* = 0.001, *η*^2^ = 0.16; Decelerations: *F* = 1.394, *p* = 0.03, *η*^2^ = 0.09). The collapsing of the data set to consider positions in isolation detailed that fullbacks and centre forwards outputted significantly higher accelerations and decelerations than any of the other positions (*p* ≤ 0.05) (centre halves, centre midfielders and wingers).

## 4. Discussion

The aim of the present study was to examine the effect of high-intensity accelerations and decelerations on performance outcomes in games rated according to win, loss or draw. Additionally, comparisons were made between the frequency of high-intensity accelerations compared to decelerations and positional outputs across 45 elite English League Two football games. Main findings from the present study indicated that there were very large differences observed between the frequency of high-intensity accelerations and decelerations regardless of match performance outcome (i.e., win, draw, lost). Additionally, significantly more high-intensity accelerations and decelerations were displayed when games were won, supporting the proposed hypothesis. Interestingly, the present study also highlighted that the frequency of high-intensity accelerations and decelerations performed by the team was reduced in periods of fixture congestion, suggesting potential fatigue effects. Finally, analysis of positional effects in a 4–3–3 formation highlighted that fullbacks and centre forwards outputted significantly more high-intensity accelerations and decelerations than any other position. Although these present findings support previous work detailing that match outcome strongly influences physical performance metrics, differences do exist in relation to positional outputs [10]. This difference may be best explained by the present study considering only one formation.

Our findings show that high-intensity decelerations were performed much more frequently than high-intensity accelerations in all matches regardless of the match performance outcome (i.e., win, draw, lost). These findings agree with previous studies that have also reported high-intensity decelerations (of varying thresholds) to be significantly higher than equivalent intense accelerations during elite football match play [12,15,19]. From a physiological perspective, these differences may be due to a greater utilisation of eccentric muscle action when decelerating, permitting higher force generation and therefore faster changes in velocity to be attained more frequently during match play [30]. The differences may also be explained through analysis of the contemporary demands of competitive match play where players frequently perform sharp, high-intensity decelerations within constrained spaces to meet tactical and technical demands [12,19]. In contrast, accelerations may be instigated from a rolling start (player changing speed whilst running) or static start, thereby influencing the rate and volume of high-intensity accelerations performed during match play [20,31]. Collectively, these findings highlight the importance of high-intensity deceleration to maximise match performance and reduce potential fatigue effects [20,31,32]. Accordingly, sport science and medicine practitioners should ensure players are adequately exposed and prepared to perform repeated high-intensity decelerations during match play. Careful consideration should be given to the monitoring of high-intensity decelerations to ensure optimal player load management.

Importantly, significantly more accelerations and decelerations were exhibited by the team when games were won compared to drawn or lost. Interestingly, however, no significant differences were found between games drawn and lost. Accordingly, the significant moderate-to-large effect size for ‘result’ seems to support a greater frequency of accelerations and decelerations being performed in winning games. These findings may be best explained by the chaotic nature of the game and the increasing demand to perform high-intensity actions over a 90-minute period when both in and out of possession in order to successfully impact the match performance outcome [25,33]. The highest difference between the frequency of high-intensity accelerations and decelerations was during losing matches. This may be due to more defensive agility actions in which high-intensity pressing or reactive braking movements may be required to quickly close down attacking players, resulting in greater high-intensity decelerations and higher magnitudes of braking forces [34]. Consideration should be given to this high exposure and how athletes are best prepared within training programming to reduce injury risk and maximise performance [35]. Coaches should be aware of how the ‘game-model’ being deployed may lead to changes in the frequency of high-intensity accelerations and decelerations and how these tactics may potentially lead to increased or decreased exposure to high-intensity accelerations and decelerations. This may have important implications for strategic periodization of team tactics and player rotations across the season and when playing against perceived higher- or lower-level opposition.

Findings within the current body of work further emphasised a moderate effect for game type. Saturday games following a mid-week fixture displayed significantly lower high-intensity accelerations and decelerations than the preceding mid-week game. Contextualising this information against the results presents some interesting findings. Notably, for Saturday games that followed a mid-week fixture, the team only won 1 game out of the 15 games that appeared in this scenario. This is a notable effect with average team high-intensity accelerations and decelerations both being shown to be significantly lower in these Saturday games. This is also observed when analysing the Saturday games that were completed in isolation and did not follow a mid-week fixture. Fixture-congested periods have been shown to have no effect on performance outcome measures such as total distance [9]; however, high-intensity variables such as accelerations and decelerations declined within the present study and therefore need to be closely analysed in the future when examining fixture congestion. Declines in high-intensity accelerations and decelerations could potentially be due to accumulative fatigue within these periods or a failure to recover from pre-game training/game exposure [22].

These considerations have important ramifications for players that lack the capacity to perform these important high-intensity actions, particularly decelerations, during fixture congested periods. For example, a reduced deceleration capacity would result in players not being able to perform sharp, suddenly imposed change of direction/speed manoeuvres, which could lead to potential detriments in team tactical goals (e.g., high press) and overall performance outcomes, whilst increasing injury risk [31,33,36]. The reduction in high-intensity accelerations and decelerations during the second match in congested calendar weeks observed in this study provides further evidence regarding the temporal effect of these actions on player fatigue. For practitioners, it highlights the importance of optimising an athlete’s preparedness for these demanding periods of the competitive season to optimise team performance. Careful consideration should be given to recovery protocols and training design to maximise physical preparedness for the upcoming fixtures. This approach, however, does not provide consideration to the opposition players and how they may affect physical performance. Literature has shown that a higher standard of opponent has shown to negatively affect the output of these variables [33]. It is unclear why this effect occurs, with some contextual suggestions indicating this may be due to higher outputs earlier on in the game, resulting in fatigue. A limitation of the present study is that it solely observed 90-minute outputs within one team over one season and has not considered gender effects on physical performance. Further research should consider quantifying how high-intensity actions such as accelerations and decelerations change within a game based on the score line. In addition to this, consideration should be given to analysing a greater number of teams across varying levels of football.

Positional analysis over the 45 games investigated in the current study identified that fullbacks and centre forwards exhibit significantly more high-intensity accelerations and decelerations than any other positions. Effect sizes for positions were highlighted as being moderate to large, indicating a strong association between high-intensity acceleration and deceleration output and position. These findings agree with Tierney et al. [37] who reported positional differences in high-intensity acceleration and deceleration outputs in an elite English football league team across a competitive season and in different formations. Furthermore, in the current study, moderate effect sizes were observed for game type. Interestingly, all positions outputted significantly higher high-intensity accelerations and decelerations in a mid-week game, with no differences when comparing Saturday games. In the current study, this aligns with the match outcome as mid-week games were also when most wins were achieved by the team (*n* = 10) and mean scores highlighted the highest frequency of high-intensity accelerations and decelerations occurred in this game type. Effect sizes for results were reported as small-to-moderate. That being said, all players exhibited significantly higher acceleration and deceleration outputs in games when the team won, again aligning with the overall team findings described. Collectively, these findings further highlight the importance of high-intensity accelerations and decelerations to match performance outcomes and the importance of closely monitoring positional differences in these metrics to ensure optimal preparation of players for forthcoming fixtures.

To our knowledge, this is the first study to specifically examine the effect of high-intensity accelerations and decelerations on match outcome. Although a variety of acceleration and deceleration thresholds have been examined previously, the significance of high-intensity accelerations (>3m/s^2^) and decelerations (<−3m/s^2^) is of particular importance [12,15]. These high-intensity actions, commonly sequenced with changes of direction, are crucial for physical performance in football [32]. They allow players to rapidly adapt to tactical demands, perform sharp movements to win dual challenges, create space with purposeful movement and defend goal attempts, all essential for team performance [32,35]. That being said, it is important to acknowledge the importance of monitoring players relative to their own physical performance capacities, and future work should consider monitoring high-intensity accelerations and decelerations using relative thresholds [38] whilst detailing any effect on physical performance or match outcome. Training practice should account for the effect these high-intensity actions have on match outcome when preparing players for the demands of competition.

## 5. Conclusions

High-intensity accelerations and decelerations are important match actions that allow players to adapt to tactical demands, win duals and create/defend goal attempts, all critical for match outcome. This is the first study to investigate the influence of high-intensity accelerations and decelerations on team performance over a competitive season in an English League Two football team. Importantly, our findings suggest there is an impact of these high-intensity actions on team performance, in that outputs were highest when the team won the game and lowest during draws or losses. Across the competitive season, players performed more high-intensity declarations than accelerations. Thus, in contemporary soccer match play particular consideration may be needed to prepare players for the demands of high-intensity decelerations. In addition, the positional demands of high-intensity accelerations and decelerations are important to consider when preparing and conditioning players for competition throughout the season. Our findings have important implications for practitioners and provide evidence for the utility of these important metrics during the competitive season to optimise team performance.

## Figures and Tables

**Table 1:** Frequency of team high-intensity (>3 m/s^2^) accelerations and decelerations (mean ± SD) for weeks associated with midweek games, Saturday games following a midweek game, Saturday games only and total games.

Fixture Details	Total Accelerations (m/s^2^)	Average Accelerations (m/s^2^)	Total Decelerations (m/s^2^)	Average Decelerations (m/s^2^)	Number of Games
Midweek Game	2772	185 ± 53	5473	365 ± 91	15
Saturday Game Following Midweek Game	2272	151 ± 36	4576	305 ± 65	15
Saturday Game Only	2475	155 ± 26	5211	326 ± 51	15
Total (all games)	7355	165 ± 43	14,866	330 ± 75	45

**Table 2:** Frequency of team high-intensity (3 m/s^2^) accelerations and decelerations (mean ± SD) for weeks and results associated with midweek games, midweek and Saturday games and Saturday-only games for one season.

Fixture Details	Result	Total Accelerations m/s^2^	Average Accelerations m/s^2^	Total Decelerations m/s^2^	Average Decelerations m/s^2^	Number of Games
Midweek Game	Win	2136	214 ± 39	5473	396 ± 92	10
	Draw	106	106 ± 10	241	241 ± 0	1
	Loss	530	133 ± 20	1269	317 ± 36	4
Saturday game following mid-week game	Win	118	118 ± 14	297	297 ± 0	1
	Draw	789	132 ± 32	1641	274 ± 55	6
	Loss	1353	169 ± 28	2638	330 ± 66	8
Saturday game with no mid-week game	Win	1076	154 ± 28	2222	317 ± 56	7
	Draw	632	147 ± 20	1277	319 ± 40	4
	Loss	579	145 ± 26	1255	314 ± 29	4
Total of all games	Win	3330	185 ± 47	6482	360 ± 87	18
	Draw	1747	146 ± 35	3487	291 ± 53	12
	Loss	2278	152 ± 30	4897	326 ± 57	15

**Table 3:** Frequency of Positional Accelerations and Decelerations (Mean ± SD) within a 4–3–3 Formation over 45 Competitive League Two Games.

	Average Accelerations m/s^2^	Average Decelerations m/s^2^
Centre Half	10.72 ± 6.20	22.96 ± 11.33
Fullback	12.34 ± 6.25	25.61 ± 15.18
Centre Midfield	9.26 ± 5.33	22.43 ± 14.44
Winger	12.96 ± 6.74	25.28 ± 15.59
Centre Forward	14.07 ± 8.14	17.75 ± 14.73

## Data Availability

Data not available due to club agreement.
